# Toxicity of fatty acid profiles of popular edible oils in human EndoC-βH1 beta-cells

**DOI:** 10.1038/s41387-020-0108-7

**Published:** 2020-01-27

**Authors:** Anna-Sophie von Hanstein, Sigurd Lenzen, Thomas Plötz

**Affiliations:** 1 Institute of Experimental Diabetes Research, Hannover Medical School, Hannover, Germany; 2 Institute of Clinical Biochemistry, Hannover Medical School, Hannover, Germany

**Keywords:** Type 2 diabetes, Malnutrition, Fatty acids

## Abstract

An inappropriate diet, particularly excessive consumption of dietary fats and oils, may have a major negative impact on beta-cell function and cause type 2 diabetes mellitus. To investigate this issue, we examined the toxicity of free fatty acid (FFA) compositions mirroring the FFA profiles of various popular edible oils in human EndoC-βH1 beta-cells and in rat islets. For this purpose, we made compositions consisting exclusively of various FFAs in different volumetric percentages mimicking these oils and additionally mixtures of these compositions. Human EndoC-βH1 beta-cells were incubated with different oil compositions and the toxicity, lipid droplet formation, ER-stress, and H_2_O_2_ production were analyzed. Compositions with prominent content of saturated as well as unsaturated long-chain FFAs showed moderate but significant toxicity both in human EndoC-βH1 beta-cells and rat islets, however, without further measurable metabolic impairments. On the other hand compositions with high content of medium-chain FFAs revealed no toxicity. A composition with 50% of the very long-chain unsaturated FFA erucic acid caused high toxicity with concomitant peroxisomal H_2_O_2_ production. The toxicity of FFAs to human EndoC-βH1 beta-cells was dampened in mixtures of FFA compositions with a significant content of medium-chain FFAs, but not with a significant proportion of unsaturated FFAs.

## Introduction

Dietary habits can have a major impact on health and may cause obesity and type 2 diabetes mellitus (T2DM). Numerous studies have examined the effects of dietary oil consumptions on human health; however, with conflicting results^1–3^. A prominent example is olive oil, containing mostly the monounsaturated free fatty acid (FFA) oleic acid, which is thought to have beneficial effects on cardiovascular health and the metabolic state in T2DM^3^. On the other hand, coconut oil, rich in medium-chain FFAs, has been promoted for positive effects on health^1^ although clinical trials could not provide solid evidence for this assumption^2^.

In general, saturated FFAs are thought to be harmful due to lipotoxicity, while unsaturated FFAs are considered to be beneficial^4^. An excessive fat intake may have a major negative impact on beta-cell function. Since individuals consume oils composed of various saturated and unsaturated FFAs, FFA mixtures represent the typical lipid component of the daily diet.

In the present study, we, therefore, compared the effects of different FFA profiles of thirteen popular edible plant oils and butter on human EndoC-βH1 beta-cells and of selected popular oils on isolated rat islets. In addition, we analyzed the toxicity of the former traditional version of old rapeseed oil with its characteristically high content of the very long-chain erucic acid (C22:1). Nowadays it has been replaced due to its toxicity by new rapeseed oil varieties with a very low erucic acid content^5^.

We generated oil compositions mimicking the FFA profiles of these edible oils containing different long-chain saturated, monounsaturated, diunsaturated, polyunsaturated and medium-chain FFAs. Since we did not include other constituents such as antioxidants, we were able to associate differences in toxicity to the different FFA species in these oils.

In a last step, we tested the toxic effects of mixtures of the FFA compositions of four different edible oils in order to elucidate whether it is possible thereby to ameliorate the toxic potential of individual edible oil compositions.

## Material and methods

### Cell culture

Human EndoC-βH1 beta-cells (ENDOCELLS SARL, Paris, France) were cultured^6^ and regularly tested for mycoplasma. Rat pancreatic islets were isolated as described^7^.

### FFA compositions mimicking edible oils

The FFA compositions contain FFAs (all from Sigma-Aldrich, Munich, Germany; C22:1 from Larodan AB, Solna, Sweden) in different volumetric percentages mimicking various popular edible oils and butter. The percentages of single FFAs for each composition were taken from^5,8–11^, adapted and listed in Table S1. Mixtures 1a-2b were composed of equal parts of the FFA compositions depicted in Table S1. Fresh 50 mM FFA stock solutions were prepared in 90% ethanol. Cells were incubated in cell culture medium containing 2% fatty acid free BSA (Serva, Heidelberg, Germany), 1% externally added BSA and a final concentration of 500 µM FFA composition, control cells were incubated with the same concentration of ethanol only.

### Experimental methods

EndoC-βH1 beta-cells (passages 3–30) and isolated rat islets (from 6 male Lewis rats of 300–400 g) were incubated with different FFA compositions for 24 or 48 h. Thereafter caspase-3 activity^12^ and for EndoC-βH1 beta-cells lipid droplet formation^13^, H_2_O_2_ generation (see Supplementary Information), as well as RT-PCR for the quantification of ER-stress marker^12^, were measured as described.

### Statistical analysis

Data are expressed as means ± SEM and tested for significance using analysis of variance (ANOVA) plus Dunnett’s multiple comparison test (Graphpad, San Diego, CA, USA).

## Results

### Toxicity of FFA compositions mimicking edible oils in EndoC-βH1 beta-cells and rat pancreatic islets

**Fig. 1:**
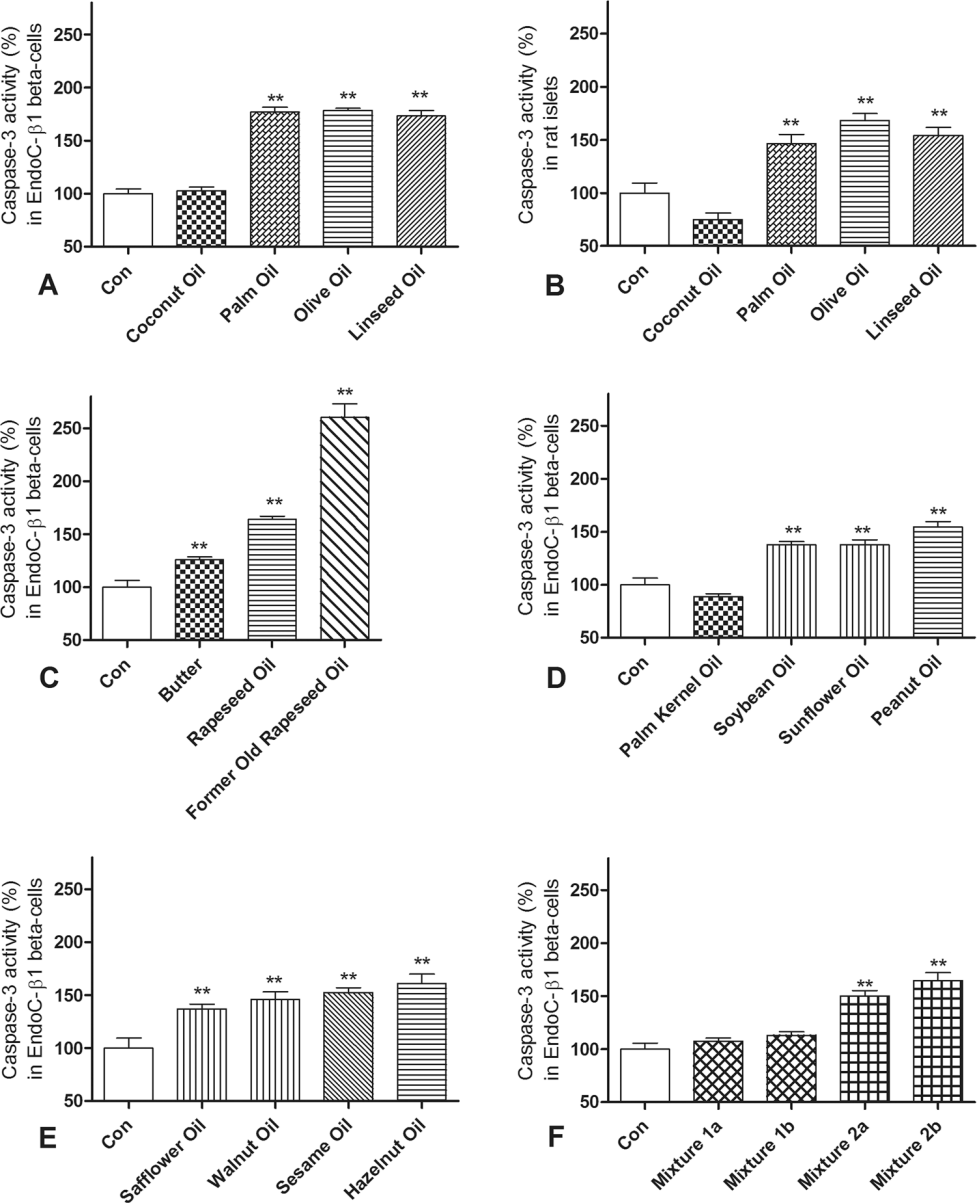
Toxicity of different FFA compositions mimicking popular edible plant oils and butter as well as selected mixtures of them in human EndoC-βH1 beta-cells and rat islets. Human EndoC-βH1 beta-cells (**a**) and isolated rat islets (**b**) were incubated for 2 days with compositions of different FFAs (total concentration 500 µM each) simulating the most popular edible plant oils. In addition EndoC-βH1 beta-cells were incubated with further FFA compositions mimicking additional edible oils and butter (**c**–**e**) as well as with mixtures composed of equal volumes of selected FFA compositions (**f**). Mixture 1a contained the FFA compositions mimicking coconut oil, olive oil, linseed oil, and palm oil while mixture 1b was composed of the FFA compositions mimicking palm kernel oil, soybean oil, sunflower oil, and safflower oil. Mixture 2a contained the FFA compositions mimicking rapeseed oil, sesame oil, peanut oil, and walnut oil whereas mixture 2b was made of the FFA compositions mimicking linseed oil, soybean oil, rapeseed oil, and walnut oil. After incubation activation of caspase-3 was measured. Data are expressed as means ± SEM of 4–9 independent experiments. **p < 0.01 compared to untreated cells (Dunnett’s Multiple Comparison Test).

To investigate the toxicity of FFA compositions mimicking edible oils we determined the activity of the toxicity marker caspase-3, starting with a group of the most important popular oils in human EndoC-βH1 beta-cells (Fig. 1a) as well as rat islets (Fig. 1b). In addition, we examined the toxicity of other FFA compositions that mimic the compositions of further different popular edible oils in EndoC-βH1 beta-cells (Fig. 1c–e).

Exposition of EndoC-βH1 beta-cells to FFA compositions with a significant content of medium-chain FFAs (C8–C14) (mimicking coconut oil, Fig. 1a and palm kernel oil, Fig. 1d) did not result in a significant induction of caspase-3 as compared to control cells. In contrast, FFA compositions containing major quantities of long-chain FFAs but no medium-chain FFAs exhibited a moderate toxic effect. This effect could be observed after exposure to oil compositions with a high content of the saturated long-chain FFA palmitic acid (mimicking palm oil, Fig. 1a) as well as compositions containing large percentages of unsaturated long-chain FFA with different numbers of double bonds, such as the monounsaturated FFA oleic acid (in olive oil, Fig. 1a; rapeseed oil, Fig. 1c; peanut oil, Fig. 1d and hazelnut oil, Fig. 1e), the diunsaturated FFA linoleic acid (in soybean oil, Fig. 1d; sunflower oil, Fig. 1d; safflower oil, Fig. 1e; walnut oil, Fig. 1e and sesame oil, Fig. 1e) and the polyunsaturated FFA α-linolenic acid (prominent in linseed oil, Fig. 1a). The toxic potential of the composition mimicking butter (Fig. 1c), with both medium-chain FFAs and long-chain FFAs, was lying in-between the two groups.

Incubation with a composition containing 50% erucic acid (“former old rapeseed oil”) resulted in a 2.5-fold increase of caspase-3 activity, which was significantly higher than the toxicity of the composition mimicking present-day rapeseed oil (Fig. 1c).

Next, we examined four mixtures created from different characteristic FFA compositions (Fig. 1f). Mixtures 1a and 1b contained compositions with medium-chain FFAs (mimicking coconut oil and palm kernel oil, respectively) among other FFA components (as shown in Table S1). They caused no significant toxicity in the human EndoC-βH1 beta-cells, in contrast to mixtures 2a and 2b, which were composed only of FFA compositions containing different long-chain FFAs, which again showed a moderate toxic effect (Fig. 1f).

The effects of FFA compositions from the group of the most important popular oils (coconut, olive, linseed, palm oil) caused comparable effects in rat pancreatic islets (Fig. 1b) as in human EndoC-βH1 beta-cells (Fig. 1a).

### Effect of FFA compositions on formation of lipid droplets

**Fig. 2:**
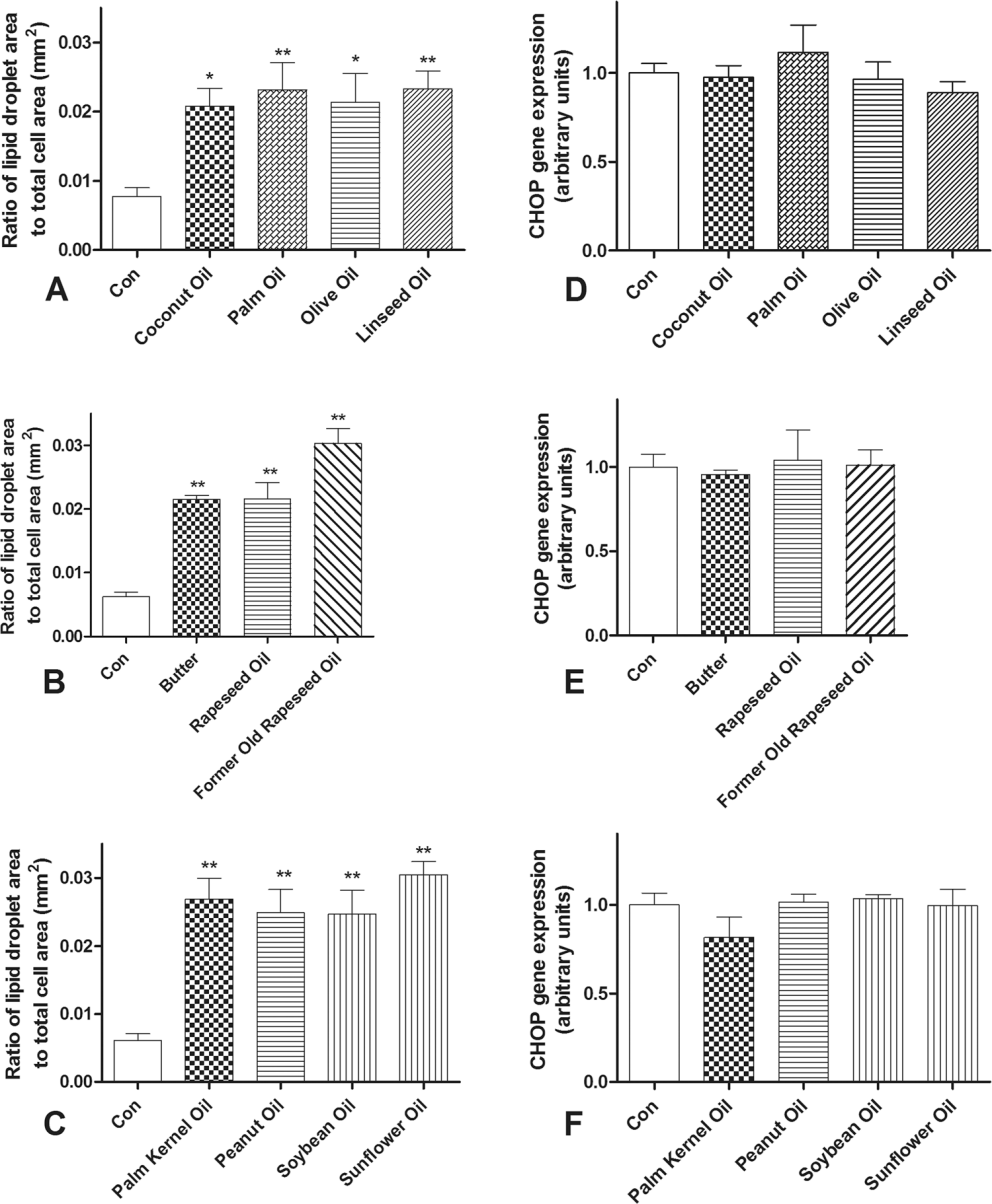
Lipid droplet formation and gene expression of ER-stress marker CHOP after incubation of human EndoC-βH1 beta-cells with different FFA compositions. Human EndoC-βH1 beta-cells were incubated with mixtures of different FFAs (total concentration 500 µM each) simulating various popular edible plant oils and butter. To measure lipid droplet formation cells were trypsinized after 48 h exposure, stained with Oil Red O and analyzed by fluorescence microscopy using the xcellence rt software. The fluorescence intensities were measured at 560/630 nm (**a**–**c**). mRNA expression of the ER-stress marker gene CHOP was quantified by RT-qPCR after 24 h exposure. Expression levels were normalized to the expression of the housekeeping genes actin, α-tubulin and TATA box binding protein (**d**–**f**). Data are expressed as means ± SEM of 4–6 independent experiments. *p < 0.05, **p < 0.01 compared to untreated cells (Dunnett’s Multiple Comparison Test).

Having shown the toxicity of different FFA compositions, we thought to gain insight into the impact of lipid droplets caused by the FFA compositions. All examined FFA compositions, containing both saturated medium-chain and long-chain FFAs together with unsaturated FFAs induced lipid droplet formation to a comparable extent in EndoC-βH1 beta-cells (Fig. 2a–c).

### Effect of FFA compositions on ER-stress

In addition, we studied the effects of the FFA compositions on ER-stress. None of the FFA compositions had an influence on the ER-stress marker CHOP in EndoC-βH1 beta-cells (Fig. 2d–e). The composition mimicking palm oil with the highest palmitic acid content (225 µM) of all FFA compositions showed also no effect on CHOP expression in rat islets (0.95 ± 0.06 as compared to 1.00 ± 0.05 in the control situation; *n* = 3).

### Effect of FFA compositions on H_2_O_2_ generation in peroxisomes and mitochondria

We then examined H_2_O_2_ generation in mitochondria and peroxisomes by organelle specific HyPer protein expression. No significant increase of H_2_O_2_ generation was observed after treatment with any of the examined FFA compositions in mitochondria (Fig. S1B), while peroxisomes showed a tendency for increased H_2_O_2_ production (Fig. S1A) and a low but significant increase after incubation with “former old rapeseed oil”.

## Discussion

The toxicity of different FFA compositions mimicking various edible oils and butter in human EndoC-βH1 beta-cells differed significantly in dependence on the chemical structure of the FFAs in the compositions.

All FFA compositions containing only long-chain FFAs (C16–C18) showed a mild but significant toxicity. When comparing the beta-cell toxicity with respect to the proportion of unsaturated FFAs in the compositions the extent of toxicity remained unchanged and was not dependent on the number of double bonds. Thus our results do not provide evidence for a major protective potential of these unsaturated FFAs within the oil compositions. Many popular oils belong to this group, including olive oil. The T2DM risk reduction through oils with a high content of unsaturated FFAs as observed in several studies^3,14^ could not be translated to human EndoC-βH1 beta-cells in our study. This does not exclude that other plant ingredients such as polyphenols might have a protective effect^15,16^. Our studies using solely FFAs showed comparable toxic effects of similar compositions. Therefore, not only the FFA profile but also the FFA source should be considered, especially for butter with its bovine origin.

On the other hand, it is obvious that FFAs with high beta-cell toxic potential must be excluded from nutritional oils. In particular since toxicity increases with increasing chain length of the FFAs^12^. The importance of an exclusion of very long-chain FFAs from nutritional oils is convincingly documented by the great beta-cell toxicity of a composition with a very high content of erucic acid (50%) as found in oils of former old rapeseed varieties.

Medium-chain FFAs shorter than C16 are not toxic to EndoC-βH1 beta-cells^7^. In the present study, we demonstrate that a high content of medium-chain FFAs (C8–C14) in compositions with long-chain FFAs mirroring the FFA profile of coconut or palm kernel oil were able to reduce toxicity of these compositions. Even when mixing FFA compositions mimicking different edible oils, the reduction of toxicity by the medium-chain FFAs could be maintained, when one of the four different edible oils in this mixture contained a high proportion of medium-chain FFAs.

The crucial toxicity observations made in human EndoC-βH1 beta-cells were confirmed also in primary rat islets.

The analyses showed increased H_2_O_2_ production in peroxisomes with concomitant moderate toxicity especially in FFA compositions with very long-chain FFAs, whereas H_2_O_2_ production in mitochondria and ER-stress were not involved. Since virtually all oil compositions caused comparable lipid droplet formation in spite of differences in their beta-cell toxicity, lipid droplet formation should not be considered a primary element responsible for the toxicity of edible oils.

## Conclusion

The toxicity of FFAs to human EndoC-βH1 beta-cells and rat islets could be dampened by mixtures of FFA compositions mimicking edible oils with a significant content of medium-chain FFAs, but not with a significant proportion of unsaturated long-chain FFAs.

## Supplementary information

Supplemetary Information Tab. S1, Fig. S1, Supplementary Method
