# Supplementary material for: Toxicity of fatty acid profiles of popular edible oils in human EndoC-βH1 beta-cells
Source: Nutr Diabetes. 2020 Jan 27;10:5. doi: 10.1038/s41387-020-0108-7 (PMC7026177; doi:10.1038/s41387-020-0108-7)
Supplement: Supplementary file 1 — Supplemetary Information Tab. S1, Fig. S1, Supplementary Method [file 41387_2020_108_MOESM1_ESM.pdf]

Supplementary Information

FFA composition	caprylic acid	capric acid	lauric acid	myristic acid	palmitic acid	palmito-leic acid	stearic acid	oleic acid	linoleic acid	α-linolenic acid	erucic acid
	**C 8:0**	**C 10:0**	**C 12:0**	**C 14:0**	**C 16:0**	**C 16:1**	**C 18:0**	**C 18:1**	**C 18:2**	**C 18:3**	**C 22:1**
Coconut Oil	7.5%	7.5%	47.5%	17.5%	7.5%		2.5%	7.5%	2.5%		
Palm Oil					45.0%		5.0%	40.0%	10.0%		
Olive Oil					15.0%	2.5%	2.5%	70.0%	10.0%		
Linseed Oil					5.0%		2.5%	17.5%	15.0%	60.0%	
Butter	10.0%	2.5%	5.0%	12.5%	22.5%	2.5%	12.5%	30.0%	2.5%		
Rapeseed Oil					5.0%		2.5%	62.5%	20.0%	10.0%	
Former Old Rapeseed Oil					5.0%		2.5%	24.0%	15.0%	11.0%	45.0%
Palm Kernel Oil	2.5%	2.5%	47.5%	17.5%	10.0%		2.5%	15.0%	2.5%		
Peanut Oil					10.0%		2.5%	57.5%	30.0%		
Soybean Oil					10.0%		5.0%	25.0%	52.5%	7.5%	
Sunflower Oil					5.0%		5.0%	30.0%	60.0%		
Hazelnut Oil					5.0%		2.5%	80.0%	12.5%		
Sesame Oil					10.0%		5.0%	42.5%	42.5%		
Walnut Oil					7.5%		2.5%	20.0%	57.5%	12.5%	
Safflower Oil					5.0%		2.5%	15.0%	77.5%		
Mixture 1a	**Coconut Oil, Olive Oil, Linseed Oil, Palm Oil**										
	2.0%	2.0%	12.0%	4.0%	18.0%	0.5%	3.0%	34.0%	9.5%	15.0%	
Mixture 1b	**Palm Kernel Oil, Soybean Oil, Sunflower Oil, Safflower Oil**										
	0.5%	0.5%	12.0%	4.5%	7.5%		4.0%	21.0%	48.0%	2.0%	
Mixture 2a	**Rapeseed Oil, Sesame Oil, Peanut Oil, Walnut Oil**										
					8.0%		3.0%	45.5%	38.0%	5.5%	
Mixture 2b	**Linseed Oil, Soybean Oil, Rapeseed Oil, Walnut Oil**										
					7.0%		3.0%	31.0%	36.0%	23.0%	

Tab. S1. FFA compositions mimicking edible oils, butter and mixtures composed of selected characteristic FFA compositions

The different volumetric percentages (1-5) of saturated, mono-, di- and polyunsaturated FFAs of the employed FFA compositions mimicking edible oils and butter are depicted. Additionally different mixtures of selected FFA compositions were prepared as shown.

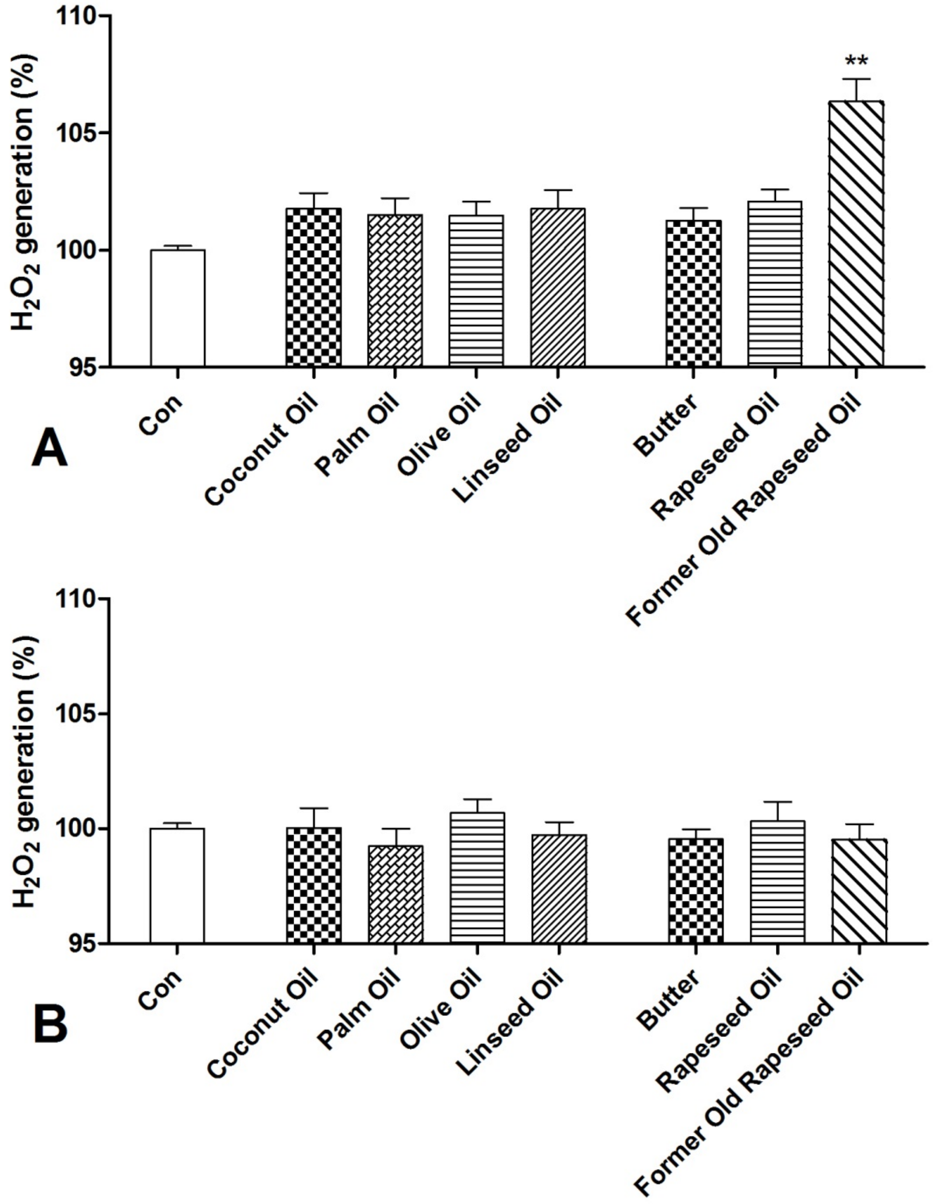

Fig. S1. Peroxisomal (A) and mitochondrial (B) H_2O_2 production in human EndoC-βH1 beta-cells after incubation with different FFA compositions

EndoC-βH1 beta-cells stably expressing the H_2O_2 sensor protein HyPer in peroxisomes (A) or mitochondria (B) were incubated for 48 h with different FFA compositions (total 500 µM each). Thereafter the fluorescence ratio was measured and normalized to control cells.

Data are means±SEM of 15-16 independent experiments. **$p<0.01$ compared to untreated cells (Dunnett's Multiple Comparison Test).

Supplementary Method

Analysis of H_2O_2 generation using HyPer proteins

EndoC-βH1 beta-cells stably expressing the H_2O_2 sensor protein HyPer in peroxisomes or mitochondria were generated as described (6) and 48 h after treatment with different FFA compositions cell imaging analyses were performed (6).

Supplementary References

1. *Codex Alimentarius*. Standard For Named Vegetable Oils. Codex STAN 210-1999. 2001; 5-6
2. *National Nutrient Database for Standard Reference* [database on the Internet]. United States Department of Agriculture. 2018 [cited Feb 2018]. Available from: https://ndb.nal.usda.gov/ndb/foods/show/1?n1=%7BQv%3D1%7D&fgcd=&sort=default&offset=&format=Full&new=&measureby=&Qv=1&ds=Standard+Reference&qt=&qp=&qa=&qn=&q=&ing=.
3. Kostik V, Memeti S, Bauer S. Fatty acid composition of edible oils and fats. *J Hyg Eng Des* 2013; **4**: 112-116.
4. Orsavova J, Misurcova L, Ambrozova JV, Vicha R, Mlcek J. Fatty acids composition of vegetable oils and its contribution to dietary energy intake and dependence of cardiovascular mortality on dietary intake of fatty acids. *Int J Mol Sci* 2015; **16**(6): 12871-12890.
5. Röbbelen G. Der gegenwärtige Stand der Züchtung von Erucasäure-, Linolensäure- und Glucosinolat-Armen Rapssorten. *Qualitas Plantarum* 1973; **23**(1): 221-238.
6. Plötz T et al. Structure-toxicity relationships of saturated and unsaturated free fatty acids for elucidating the lipotoxic effects in human EndoC-betaH1 beta-cells. *BBA Mol Basis Dis* 2019; **1865**(11): 165525.
